# Impact of Lens Thickness on Outcomes After Cataract Versus Combined Cataract–Glaucoma Surgery in a Predominantly Black Population

**DOI:** 10.3390/jcm15135111

**Published:** 2026-07-01

**Authors:** Devin Giordano, Jasmine Okafor, Daniel Laroche

**Affiliations:** 1 Advanced Eyecare of New York, New York, NY 10027, USA; jasmineokafor0@gmail.com (J.O.); dlarochemd@aol.com (D.L.); 2 School of General Studies, Columbia University in the City of New York, New York, NY 10027, USA

**Keywords:** cataract, glaucoma, lens thickness, cataract surgery, intraocular pressure

## Abstract

**Background/Objectives**: We aimed to evaluate the relationship between lens thickness (LT) and postoperative outcomes following cataract surgery versus combined cataract–glaucoma procedures in a predominantly Black and Caribbean population, and to assess the utility of LT and the Laroche Glaucoma Risk Calculator in predicting intraocular pressure (IOP) reduction. **Methods**: This retrospective cohort study included 187 eyes from patients aged ≥50 years that underwent cataract surgery alone or combined cataract–glaucoma surgery (goniotomy or Ahmed retrobulbar/intraconal tube) at a single center in Queens, New York. Preoperative and ≥3-month postoperative data included IOP, visual acuity (logMAR), medication burden, visual field mean deviation, and anterior segment biometry. Patients were stratified by surgical type, diagnosis, and glaucoma risk. Associations between LT and postoperative IOP reduction were analyzed. **Results**: Mean LT was 4.53 mm. Greater LT was associated with increased postoperative IOP reduction across all groups. Eyes with LT ≥4.5 mm showed greater IOP reduction compared to LT ≤4.2 mm (2.63 vs. 1.19 mmHg). Combined procedures yielded greater IOP reduction than cataract surgery alone, with the largest decrease in the Ahmed group (−4.56 mmHg). Cataract surgery alone produced smaller but significant reductions (−1.58 mmHg) and the greatest visual acuity improvement. Medication burden decreased substantially in the combined groups. Patients with angle-closure glaucoma had the highest LT. High-risk patients demonstrated greater IOP reduction than low-risk patients. **Conclusions**: Increased LT may serve as a predictive biomarker for postoperative IOP reduction. Incorporating LT and the Laroche Glaucoma Risk Calculator into preoperative planning may enhance surgical decision-making and outcomes, particularly in underserved populations.

## 1. Introduction

A possible correlation between lens thickness (LT) or other ocular biometric parameters and IOP (intraocular pressure) reduction following cataract surgery or combined cataract and glaucoma surgery has been proposed by current literature [1,2]. Additionally, patients with primary angle closure or those categorized as primary angle-closure suspects tend to show a greater IOP reduction following phacoemulsification than open-angle and non-glaucomatous groups [3]. It is well documented that cataract surgery, especially earlier intervention, can reduce IOP and slow the progression of glaucoma or help prevent it in patients who are at high risk [4]. As increased lens thickness is associated with angle-closure glaucoma and aging, more analysis on the correlation between LT and surgical outcomes in different populations, especially in an aging society, might illuminate LT analysis as a novel tool in predicting surgical outcomes and planning a timeline for surgical intervention [5]. The EAGLE trial demonstrated that early clear lens extraction was superior to laser peripheral iridotomy for IOP control in primary angle closure, directly supporting the mechanistic rationale that removing a thicker lens improves aqueous outflow [6].

Previous studies have shown that African patients have significantly smaller lens thickness than White patients, which may have implications for angle-closure risk and IOP response after cataract surgery. This directly supports the need for population-specific biometric data [7]. There is a lack of research concerning ocular biometric parameters, including LT, in Black and Caribbean populations [8].

The Laroche Glaucoma Risk Calculator is a useful tool that we use as initial screening for glaucoma by checking IOP, CCT, and age. This paper aims to determine the predictive value of lens thickness in surgical outcomes (IOP, medication burden, logMAR, and visual field defect) across surgery types (cataract versus combined cataract and glaucoma; Ahmed retrobulbar/intraconal tube surgery versus goniotomy) in the majority of Black and Caribbean population. We also investigated how patient risk with the Laroche calculator and lens thickness were correlated.

## 2. Materials and Methods

This study was approved by the BRANY Institutional Review Board (Protocol 221382371543). A waiver of informed consent was granted under 45 CFR 46.116(d) due to the retrospective nature of the study, as the research involved no more than minimal risk to participants; the waiver did not adversely affect the rights and welfare of subjects, and the research could not practicably be carried out without the waiver. The study was a retrospective cohort analysis using existing data obtained from a single-center private practice ophthalmology in Queens, New York. The anterior chamber biometric data were obtained (Bausch + Lomb SeeNa) during patients’ routine surgical consultation visits. Other data, such as pre- and post-operative IOP, logMAR, visual field defect, medication burden, demographic information, and diagnosis/risk status, were collected from the practice’s electronic health record.

Inclusion criteria included being 50 years or older at the time of surgery, undergoing either cataract or combined cataract and glaucoma surgery, and having completed a follow-up appointment of at least 3 months after surgery. A total of 187 eyes fell into the inclusion criteria and were subsequently stratified by surgery type and diagnosis.

The statistical approach used in this study included paired *t*-tests for within-group IOP comparisons and multivariable linear regression adjusting for key covariates such as baseline IOP, age, diagnosis, surgical type, and medication burden. The inclusion of interaction terms and reporting of confidence intervals alongside *p*-values was noted. (Table 1).

## 3. Results

Baseline characteristics differed across the three surgical groups (cataract alone, cataract–goniotomy, and cataract–Ahmed). Mean preoperative visual acuity (logMAR) was worst in the cataract-alone group (0.70), compared to 0.58 in the cataract + goniotomy group and 0.44 in the cataract + Ahmed retrobulbar group. In contrast, mean preoperative intraocular pressure (IOP) was lowest in the cataract-alone cohort (14.74 mmHg) and higher in the combined procedure groups (17.32 mmHg for goniotomy and 18.56 mmHg for Ahmed retrobulbar). Similarly, medication burden increased across groups, with no preoperative medications in the cataract-alone group versus 2.42 and 3.33 medications in the goniotomy and Ahmed retrobulbar groups, respectively. Among patients undergoing combined procedures, baseline visual field loss was more advanced in the Ahmed retrobulbar group (mean deviation −18.28 dB) compared to the goniotomy group (−3.81 dB), suggesting more severe disease in this cohort at presentation. (Table 2).

Postoperative outcomes demonstrated improvement across all groups, with the magnitude of change varying by intervention. Visual acuity improved in each cohort, with the greatest gain observed in the cataract-alone group (−0.52 logMAR), followed by the cataract + goniotomy (−0.37) and cataract + Ahmed retrobulbar groups (−0.15). Intraocular pressure (IOP) reduction was modest in the cataract-alone group (−1.58 mmHg, 11%) but more pronounced in the combined procedure groups, particularly in the Ahmed retrobulbar group (−4.56 mmHg, 25%), compared to the goniotomy group (−2.66 mmHg, 16%). Medication burden remained unchanged in the cataract-alone group but decreased substantially in both combined groups, with an 80% reduction in the goniotomy group and a 47% reduction in the Ahmed retrobulbar group. Visual field mean deviation remained relatively stable postoperatively, with a slight decline in the goniotomy group (−0.94 dB) and minimal improvement in the Ahmed retrobulbar group (+0.47 dB). Overall, combined procedures were associated with greater reductions in IOP and medication use, while visual acuity improvements were observed across all groups. (Table 3).

The mean lens thickness across all patients was 4.53 mm (n = 187). Subgroup analysis demonstrated variation by diagnosis and surgical intervention, with patients with angle-closure glaucoma undergoing Ahmed retrobulbar surgery exhibiting the greatest mean preoperative lens thickness (4.81 mm, n = 7). Patients with open-angle glaucoma had a mean lens thickness of 4.51 mm (n = 42), while those undergoing cataract surgery alone had mean values of 4.57 mm (n = 55) in the high glaucoma risk group and 4.49 mm (n = 66) in the low-risk group. Across all subgroups, scatter plot analysis revealed a consistent positive association between increasing lens thickness and greater postoperative intraocular pressure reduction. (Table 4).

**Figure 1:**
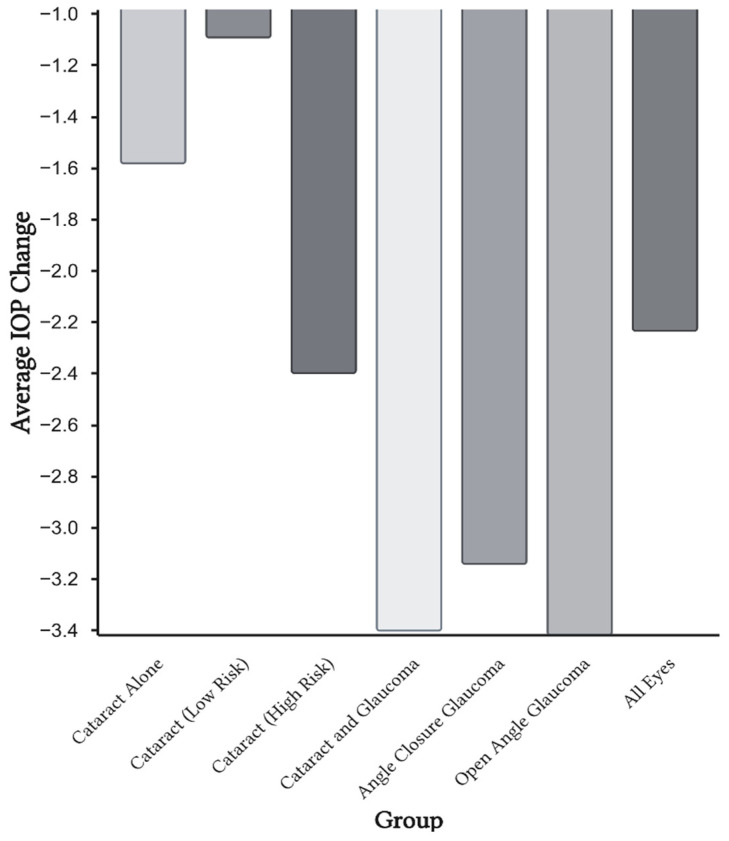
IOP change versus group.

Eyes with an LT of 4.5 mm or greater had an average IOP reduction of 2.63 ± 4.59 mmHg, while eyes with an LT of 4.2 or lower had an average reduction of 1.19 ± 4.10 mmHg. Stratification of the cataract-alone cohort using the Laroche Glaucoma Risk Calculator demonstrated significant postoperative IOP reduction in both risk groups. The high-risk group showed a greater mean reduction of 2.4 mmHg from a baseline of 15.73 mmHg (paired t-test, *p* < 0.0001; 95% CI: 1.40–3.40), while the low-risk group experienced a smaller but still significant reduction of 1.09 mmHg from a baseline of 14.19 mmHg (*p* ≈ 0.0002; 95% CI: 0.54–1.64). Overall, the magnitude of IOP lowering was more pronounced in the high-risk group. In comparisons of combined procedures, both cataract–goniotomy (*p* ≈ 0.002) and cataract–Ahmed groups (*p* ≈ 0.003) demonstrated significant postoperative IOP reductions. Although the Ahmed group achieved a larger mean reduction than the goniotomy group (−4.56 vs. −2.66 mmHg), this difference did not reach statistical significance on between-group analysis. (Figure 1).

In multivariable linear regression adjusting for baseline IOP, age, surgical type, glaucoma diagnosis, and medication burden, lens thickness demonstrated a positive but non-significant association with postoperative IOP reduction (β = 0.74 mmHg per mm increase, *p* = 0.228). Baseline IOP was the strongest independent predictor of IOP reduction (β = 0.696, *p* < 0.0001). Age was also independently associated with greater IOP reduction (*p* = 0.020). Several glaucoma subtypes demonstrated differential responses to surgery, indicating heterogeneity in treatment effect across diagnoses.

In a multivariable linear regression model including an interaction term between lens thickness and surgical type, a significant interaction was observed (β = 2.49, *p* = 0.045), indicating that the effect of lens thickness on IOP reduction differed by surgical group. Specifically, lens thickness was not associated with IOP reduction in cataract surgery alone (β = −0.086, *p* = 0.898), but demonstrated a significant positive association in combined cataract–glaucoma procedures.

In a simplified model adjusting for baseline IOP and surgical type, lens thickness emerged as a significant independent predictor of IOP reduction (β = 1.17 mmHg per mm increase, *p* = 0.035).

Stratified analyses further supported these findings. In the cataract-alone group, lens thickness was not associated with IOP reduction (*p* = 0.795), whereas in the combined surgery group, a positive association was observed (β = 2.21), though this did not reach statistical significance (*p* = 0.104), likely due to limited sample size.

## 4. Discussion/Conclusions

This study demonstrates that lens thickness (LT) may serve as a meaningful biomarker associated with intraocular pressure (IOP) reduction following cataract and combined cataract–glaucoma surgery in a predominantly Black and Caribbean population. Across all surgical and diagnostic subgroups, greater LT was consistently associated with larger postoperative IOP reduction, supporting prior hypotheses that lenticular anatomy contributes to anterior segment crowding and aqueous outflow dynamics. Patients with angle-closure glaucoma (ACG), particularly those undergoing Ahmed retrobulbar procedures, exhibited the highest mean LT, aligning with established pathophysiology linking increased lens thickness to angle narrowing. Patients with a higher risk score of 6 or greater on the Laroche Glaucoma Risk Calculator also had a greater IOP lowering with thicker lenses. Age and body mass index are important predictors of increasing lens thickness. Age is captured in the risk calculator.

Importantly, the observed association between increased LT and greater IOP reduction may reflect a mechanism whereby removal of a thicker crystalline lens leads to expansion of Schlemm’s canal and improved trabecular outflow. This effect may be especially relevant in patients with angle closure, and thus a higher LT may indicate greater benefit from early cataract surgery. Additionally, stratified analysis showed that patients with thicker lenses (≥4.5 mm) experienced greater IOP reduction compared to those with thinner lenses, further supporting LT as a potential predictive parameter.

Although lens thickness did not remain statistically significant after multivariable adjustment, the direction and magnitude of the association were consistent with our hypothesis and prior mechanistic understanding. The attenuation of effect likely reflects collinearity between lens thickness and established predictors such as age and glaucoma subtype, particularly angle-closure disease.

Importantly, unadjusted and subgroup analyses demonstrated a consistent positive association between increased lens thickness and greater IOP reduction, suggesting that LT may still serve as a clinically relevant biomarker, particularly in specific surgical or diagnostic subgroups.

These findings highlight the complexity of isolating independent biometric predictors in heterogeneous glaucoma populations and suggest that lens thickness may exert its greatest predictive value in conjunction with other anatomical and clinical factors.

This study also addresses an important gap in the literature by focusing on a majority Black and Caribbean population, groups that are disproportionately affected by glaucoma yet underrepresented in biometric and surgical outcomes research. The findings suggest that LT in this population may differ from the currently accepted LT average and that it could be incorporated into preoperative assessment to help guide surgical planning and timing, particularly in high-risk patients.

This study also demonstrates for the first time that the Laroche Glaucoma Risk Calculator provides preliminary evidence that it can stratify patients by expected IOP reduction after cataract surgery, a novel application beyond its original validation as a screening tool. The finding that high-risk patients achieved significantly greater IOP reduction (2.4 mmHg vs. 1.09 mmHg, *p* < 0.0001 vs. *p* ≈ 0.0002) in the cataract-alone group and higher in the cataract and glaucoma surgery group aligns with established principles that higher preoperative IOP predicts greater absolute reduction. However, no prior study has used this simple two-parameter tool (IOP, CCT, age) to identify surgical responders to prevent glaucoma onset, lower IOP, and reduce medication burden in patients undergoing uncomplicated cataract surgery or combined cataract–glaucoma interventions. LT may serve as a complementary biomarker when combined with the Laroche calculator to help identify patients more likely to benefit from IOP lowering, while recognizing that further prospective validation is needed.

Our findings contradict Coh et al. (2016), who found that lens position parameters predicted IOP reduction in nonglaucomatous but not glaucomatous eyes [9]. Perez et al. (2019) found that in their best predictive formula, LT had a negative coefficient (−0.42) when combined with other variables, suggesting the relationship is more complex than a simple positive association [10]. Our findings also contradict a study by Rees et al. that showed that increased LT was associated with IOP spikes on postoperative day 1 in glaucoma patients [11].

The study’s focus on a predominantly Black and Caribbean population represents a meaningful strength, particularly in light of well-documented disparities in cataract care. Recent findings from the All of Us cohort indicate that non-Hispanic Black patients undergo cataract surgery at lower rates (adjusted HR 0.88 compared to White patients), while data from the SOURCE database identify Black race as an independent predictor of postoperative complications [12,13]. In light of these disparities, the present study’s findings potentially position LT and Larohe glaucoma calculator-guided surgical planning as a potential strategy to improve surgical outcomes in higher-risk glaucoma suspects and glaucoma patients in underserved populations.

Limitations include the retrospective design, relatively small sample sizes in certain subgroups (notably ACG with Ahmed), and potential selection bias inherent to a single-center study. Additionally, follow-up duration was limited to a minimum of three months, which may not capture long-term IOP trends or disease progression. Higher baseline IOP also contributes to the lowering of the IOP with regression to the mean. Future prospective studies with larger cohorts and longer follow-up are warranted to validate these findings and further elucidate the role of LT in surgical decision-making.

Thus, lens thickness appears to be positively associated with postoperative IOP reduction across cataract and combined glaucoma surgeries, suggesting its potential role as a predictive biomarker in surgical planning. Combined procedures, particularly those involving Ahmed retrobulbar implantation, resulted in greater IOP reduction and medication burden decrease, while cataract surgery alone provided meaningful IOP lowering, especially in high-risk patients. These findings support the integration of ocular biometric parameters such as LT into preoperative evaluation, particularly in high-risk and underserved populations, to optimize individualized surgical strategies and improve glaucoma management outcomes.

## Figures and Tables

**Table 1:** Population characteristics.

Variable	Characteristic	Frequency (*N* = 187)	Percent
Age (years)	50–59	28	15.0
	60–69	75	40.1
	70–79	63	33.7
	80–89	21	11.2
Sex	Female	102	54.5
	Male	85	45.5
Race	Black or Afro-Latino	154	82.4
	Asian	28	15.0
	White or Caucasian	5	2.7
Surgery Type	Cataract Alone	120	64.2
	Combined Cataract and Glaucoma	67	35.8
Diagnosis for the Cataract-Alone Group (*N* = 120)	Non-suspect for Glaucoma	65	54.2
	Suspect of Glaucoma	55	45.8
Glaucoma Surgery Type (*N* = 67)	Ahmed Retrobulbar Tube	27	40.3
	Goniotomy	38	56.7
	Hydrus Stent	2	3.0
Diagnosis for Combined Cataract and Glaucoma Group (*N* = 67)	Primary Open Angle Glaucoma	42	62.7
	Ocular Hypertension	6	9.0
	Acute Angle-Closure Glaucoma	2	3.0
	Chronic Angle-Closure Glaucoma	12	17.9
	Low Tension	2	3.0

Note: Percentages for “Diagnosis for Cataract-Alone Group”, “Glaucoma Surgery Type”, and “Diagnosis for Combined Cataract and Glaucoma Group” were calculated using the frequencies of the “Cataract Alone” and “Combined Cataract and Glaucoma” groups, respectively.

**Table 2:** Baseline measurements.

	Group		
Mean Pre-Op Parameter	Cataract Alone	Cataract–Goniotomy	Cataract–Ahmed
VA (logMAR)	0.70	0.58	0.44
IOP (mmHg)	14.74	17.32	18.56
Medications	-	2.42	3.33
VF MD (dB)	-	−3.81	−18.28

Note: Abbreviations: VA = visual acuity; IOP = intraocular pressure; VF MD = visual field mean deviation.

**Table 3:** Post-op outcomes.

	Group		
Mean Pre-Op Parameter	Cataract Alone	Cataract–Goniotomy	Cataract–Ahmed
VA (logMAR)	0.18 (−0.52)	0.21 (−0.37)	0.29 (−0.15)
IOP (mmHg)	13.16 (−1.58, 11%)	14.66 (−2.66, 16%)	14.00 (−4.56, 25%)
Medications	-	0.50 (−1.92, 80%)	1.44 (−1.89, 47%)
VF MD (dB)	-	−4.75 (−0.94)	−17.81 (+0.47)

Note: In parentheses next to the values is the change in mean value from pre-operative metrics.

**Table 4:** Lens thickness by group.

Group	Mean Lens Thickness
Overall Cohort (*N* = 187)	4.53
ACG and Ahmed (*N* = 7)	4.81
Open Angle (*N* = 42)	4.51
Cataract Alone (High Risk) (*N* = 55)	4.57
Cataract Alone (Low Risk) (*N* = 66)	4.49

Notes: Abbreviations: ACG = Angle-Closure Glaucoma; High/Low risk indicated high or low risk based on the Laroche Glaucoma Risk Calculator, which factors in CCT, IOP, and age to determine risk of glaucoma.

## Data Availability

The original contributions presented in this study are included in the article. Further inquiries can be directed to the corresponding author.
